# Slow Breathing Exercise with Multimodal Virtual Reality: A Feasibility Study

**DOI:** 10.3390/s21165462

**Published:** 2021-08-13

**Authors:** Kun-Chan Lan, Che-Wei Li, Yushing Cheung

**Affiliations:** 1 Department of Computer Science and Information Engineering, National Cheng Kung University, Tainan 701, Taiwan; klan@csie.ncku.edu.tw; 2 EBeam Operation Division, Taiwan Semiconductor Manufacturing Company, Hsinchu 300, Taiwan; cwleezv@tsmc.com.tw; 3 Smart Microsystems Technology Center, Industrial Technology Research Institute, Tainan 709, Taiwan

**Keywords:** slow breathing training, virtual reality, multimodal biofeedback

## Abstract

Many studies have shown that slow breathing training is beneficial for human health. However, several factors might discourage beginners from continuing their training. For example, a long training period is generally required for benefit realization, and there is no real-time feedback to trainees to adjust their breathing control strategy. To raise the user’s interest in breathing exercise training, a virtual reality system with multimodal biofeedback is proposed in this work. In our system, a realistic human model of the trainee is provided in virtual reality (VR). At the same time, abdominal movements are sensed, and the breathing rate can be visualized. Being aware of the breathing rate, the trainee can regulate his or her breathing to achieve a slower breathing rate. An additional source of tactile feedback is combined with visual feedback to provide a more immersive experience for the trainees. Finally, the user’s satisfaction with the proposed system is reported through questionnaires. Most of the users find it enjoyable to use such a system for mediation training.

## 1. Introduction 

Breathing is the most vital function for the sustenance of life [1]. Effective breathing is essential to maintain good health. In traditional Eastern culture, the practice of slow breathing exercise has a very long history. As shown in many studies [2,3], the habit of controlling one’s breath, usually with meditation, can bring a positive impact on heath. Moreover, a good control of breathing [2,4,5,6] has been demonstrated as a key factor of controlling blood pressure, enhancing baroreflex sensitivity, and relieving anxiety. Some prior work [7,8] showed that slow breathing exercises over three consecutive months can improve autonomic functions. For example, slow abdominal breathing combined with biofeedback can be an effective intervention for prehypertension [9].

Achieving slow breathing needs regular respiratory exercises which can improve human attention, cortisol levels, and general psychological health [10]. However, for many novices, it can often be difficult to maintain a regular practice for various reasons such as lack of time, no progress feedback, and boringness. The practice of meditation requires sustained attention that is often hard to achieve for the novices, which results in that many of them drop out from their mediation practices. The respiratory exercises, usually performed during meditation training, are normally carried out without any feedback. On the other hand, many studies [11] have shown that machine-assisted biofeedback can be useful as an aid for meditation by improving concentration and emotion regulation.

On the other hand, biofeedback is a common way to help the individual gain voluntary control over the respective physiological process and induces favorable changes. While common implementations of biofeedback make use of numerical indicators and charts to present the feedback parameters, some prior studies [12,13] have shown, by providing a virtual nature environment (e.g., a virtual avatar that mirrors the user’s action, in our case), a VR-based implementation of biofeedback can provide the user with the feeling of actually being inside the virtual environment [14] that leads to greater focus and less mind wandering because the user’s attention is involuntarily drawn toward the engaging virtual environment [15]. Such a positive effect is subject to the so-called Attention Restoration theory [16].

In this work, a slow breathing training system based on virtual reality (VR) with multimodal (visual and tactile) feedbacks is proposed to motivate a user to engage in a regular practice of breathing control. In our system, we simulate the increase in body temperature, which normally can only be felt by experienced meditators, through a tactile feedback [17] to the user. The sensed abdominal undulation is transformed into a visual feedback in the VR to guide the user for self-regulation of breathing rate. Our experiment results show that the users can reduce their breathing rate to as low as three breaths per minute (after eight sessions) with the aid of the proposed multimodal VR system. In addition, based on the questionnaire answered by the users, it is more enjoyable to practice meditation with our biofeedback system as compared to the traditional way of practicing slow breathing without any feedback.

The remainder of this paper is organized as follows. Section 2 discusses the related work. We describe our methods in Section 3. Section 4 discusses our experiments and results. Some observations are presented in Section 5. Finally, we conclude this paper in Section 6.

## 2. Related Work

### 2.1. Breathing Control Training

Many previous studies [18,19,20,21] have shown that the control of breathing during the practice of meditation improves health of people without any side effects [1,2,3,4,5,6,7,8,9,10,22,23]. For example, in a prior study [7], a total of 60 subjects were randomly divided into two groups: a slow breathing group (that practiced slow breathing exercise) and fast breathing group. After practicing for a period of three months, the increased parasympathetic activity and decreased sympathetic activity were observed in slow breathing group. Some other papers [1,6] showed that slow breathing with equal inspiration and expiration is the best technique to increase cardiac-vagal baroreflex sensitivity (BRS), improve oxygen saturation, and lower blood pressure. However, in most of these prior works, no feedback is used as a guide for control of breathing by the trainees. Therefore, the trainees have no cue of proficiency of practices. Here, we propose a VR-based biofeedback system to train people for controlling their breathing rates, which is similar to some prior work [1,2,3,5,6,9]. Nevertheless, our system is different from these studies in the following aspects: we employed multimodal feedback, including visual and tactile feedback, to improve immersive VR experiences. In addition, a questionnaire was conducted to understand the users’ satisfaction and willingness to continue using such a system for the long-term practice of meditation.

### 2.2. Respiratory Feedback

Biofeedback is a technique, using a device to convert raw physiological signals into meaningful representation, to control aspects of physiology for improving physical and emotional well-being. With that, people can be aware of their own thoughts, feelings, and behaviors regarding their physiology [11]. Several different types of biofeedback signals, such as EEG, GSR, PPG, have been adopted in previous studies, as shown in Table 1.

While these studies are similar to our proposed approach in that they employ respiratory feedback for various applications, none of them considered multimodal feedback through virtual reality. In addition, none of these works evaluated the satisfaction of the users in using the proposed system for breathing training. For example, the virtual reality system developed in [28] is similar to our system in which they also employed respiratory feedback through VR. However, our approach differs from [28] in that our virtual reality system is complemented with a realistic human model and we provide additional tactical feedback to the trainees. In contrast to most of these prior works, in which only one training session was organized, we conducted eight consecutive training sessions for a period of two weeks.

### 2.3. Virtual Realty

Virtual reality (VR) technology has been applied to many different areas, such as education [29,30], sports [31,32], civil engineering [33], automotive industries [34], and healthcare [35,36,37]. Some studies [38] have shown that providing multimodal feedback in VR can create better immersive experiences for the users. For example, a VR system with visual, auditory and tactile feedback [38] is used by patients with phantom limb to reduce the pain.

Inspired by these prior studies, in this work, we provide visual and tactile feedback through VR for breathing control. We also evaluated the effectiveness of our proposed system after conducting eight training sessions.

## 3. Method

### 3.1. System Architecture

**Figure 1:**
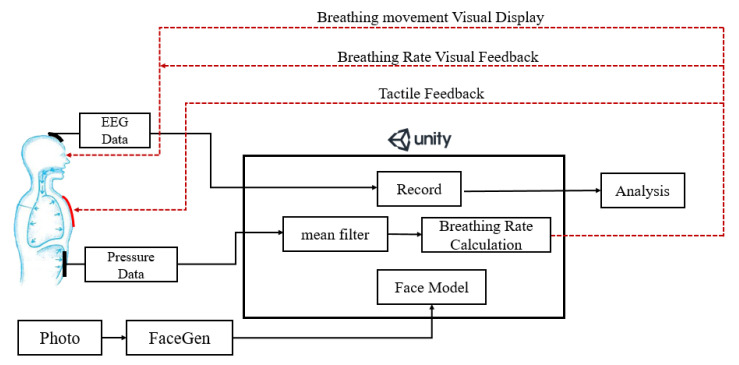
The architecture of the proposed multimodal feedback system.

The proposed multimodal feedback system and its components are shown in Figure 1. A Unity 3D [39] engine was used to create the VR App and visualize the changing of breathing rate which is detected by a pressure sensor attached to the abdomen of the user. We employed a tool called FaceGen [40] to generate a 3D human face for simulating the user who is performing the breathing training. A heating pad was sewn into the clothes of the user to simulate the rising body temperature which is usually experienced by well-trained mediators when they enter a deep state of meditation [17]. Different electric currents, according to different breathing rate, are applied to the heating pad to produce different temperatures (i.e., a higher current is applied at a slower breathing rate). Electroencephalography (EEG) data of the user were also recorded to observe the degree of concentration during the breathing training [28,41]. The details of these components are described in the next few sections.

### 3.2. Hardware

In our system, we utilized the following hardware components, including a VR headset, EEG and pressure sensors, a micro-controller and a custom-made T-shirt.

#### 3.2.1. VR Headset

**Figure 2:**
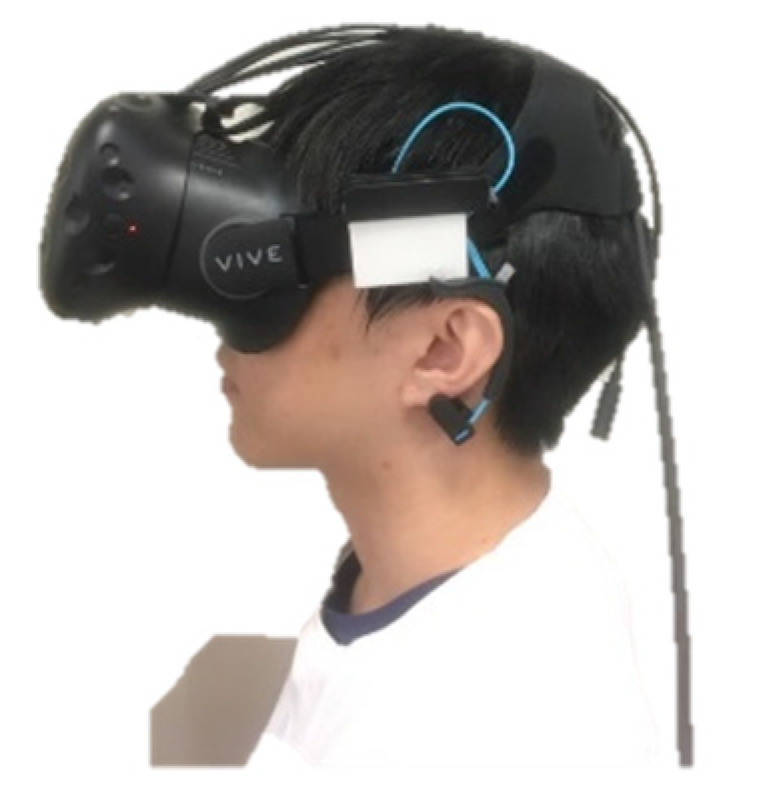
The VR HMD with the attached EEG sensor.

We used the HTC Vive (HTC Corporation, New Taipei Taiwan), which covers a nominal field of view of about 110 degrees (approximately 90 degrees per eye, with overlapping segments). Vive provides a resolution of 1080 × 1200 pixels per eye (2160 × 1200 pixels in total). In order to mount Mindwave on the VR headset for the ease of data collection, we modified the Mindwave so that it can be put into a small box which is then attached to the headset, as shown in Figure 2.

#### 3.2.2. EGG Sensor

To understand how the proposed VR-aided breathing training affects the brain activity, we utilize Neurosky Mindwave Mobile+ [42], which is a single-channel EEG sensor, to collect the EEG signals wirelessly (through Bluetooth). In our experiments, we attached the sensor to the Fp1 position of the subject. MindWave provides a sampling rate of 512 Hz and a resolution of 12 bits. The device applies a 3–100 Hz bandpass filter to EEG signals before transmitting them over Bluetooth. MATLAB (R2018B) (MathsWork, Natick, MA, USA)was used for processing these data. The first and last 30 s of data (from the 20 min training session) were removed to reduce data noise. The time bin was set to one second (i.e., a total of 1140 time bins in 19 min). These time bins were processed using FFT to obtain the band power of different frequencies.

#### 3.2.3. T-Shirt with a Heating Pad and a Pressure Sensor

**Figure 3:**
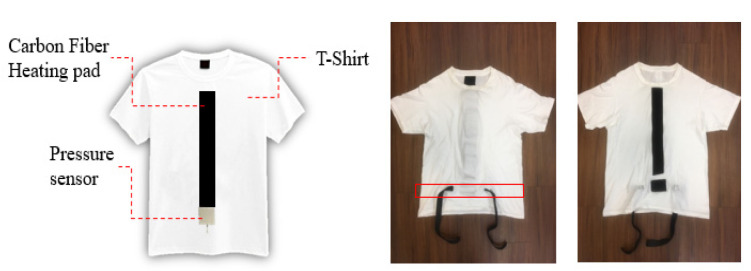
(**Left**) Sketch map of the modified T-shirt. (**Right**) Appearance from both sides of the T-shirt.

As shown in Figure 3, two (long and short) Velcro tapes were sewn into the inner parts of the clothes. A heating pad was attached with long Velcro tape, while a pressure sensor was attached to the short Velcro tape. Two buckles, used to tie the pressure sensor onto the abdomen, were sewn into the outer parts of the clothes.

#### 3.2.4. Computation Module

**Figure 4:**
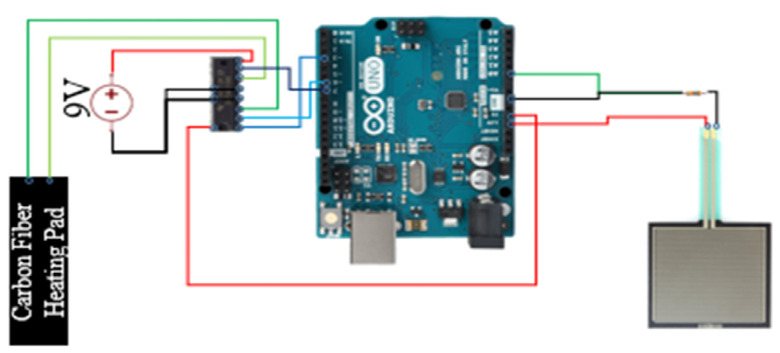
The architecture of the computation module.

As shown in Figure 4, Arduino UNO (Arduino, Boston, MA, USA)was used as the computation unit which communicates with a PC via UART (Universal Asynchronous Receiver/Transmitter). A square pressure sensor (FSR-406)(Interlink Electronics, Camarillo, CA, USA) was used to detect the abdominal movements due to the breathing. Based on FSR-406 data sheet [43], we were able to estimate the actuation force according to the output voltage of the pressure sensor. The breathing rates were calculated on board and then fed to the Unity3D (Unity Technologies, San Francisco, CA, USA) engine that runs on the PC to create visual feedback. UNO supplies 5 Vdc power to the pressure sensor with a 16 K resistor and the sampling rate was set to 200 Hz. In addition, UNO provides 3.3 Vdc to power a heating pad (sewn into a T-shirt) with a 10Ω piece of carbon fiber connected to a variable resistor (SN754410) (Texas Instruments, Dallas, TX, USA). The variable resistor was used to regulate the electric current based on detected breath rates. An external power supply (Keysight E365A) (Keysight Technologies, Santa Rosa, CA, USA) was used to provide 9 Vdc to power this system.

### 3.3. Sensor Data Collection and Visualization

Meditative visualization is a technique commonly used in medication by generation and maintenance of certain mental images, which helps the meditator quickly enter a state of deep concentration and relaxation [44]. For example, when practicing an ancient meditation technique called g-tummo [45], the meditator is required to visualize a ‘channel’ going from his perineum to the head. When he starts practicing slow breathing, the breath energy will ignite the “inner fire” in this channel. However, given that performing such a guided imagery could be difficult to a meditation beginner, in this work, we utilize VR to create a virtual avatar that mirrors the user’s behavior (e.g., slow breathing) and an imagery of “firing channel”.

Such visualization is realized by Unity3D which consists of scenes, models, and scripts. Here, models (objects) are created in the scenes and scripts are programmed to control the behavior of the models. In this study, breathing data were collected and input into Unity3D to visualize the abdominal movements (that reflects the breathing) and changing body temperatures (during the creation of the “inner fire”).

#### 3.3.1. Breathing Data

The pressure sensor is sampled at a rate of 200 Hz. A mean filter is applied for every 10 data points from the sensor, an average of those data points is calculated to produce a smoother signal. To detect peaks in this pressure wave signal (which is considered as the points of maximum inhalation), we implemented a threshold-based algorithm to detect the peak based on a simplified version of an event-related moving average method [46] which consists of three stages: signal pre-processing (including bandpass filtering), the generation of blocks of interest using two moving averages, and adaptive thresholding. Specifically, we first applied a 1 Hz low-pass filter to remove high frequencies that do not contribute to the peak. Given that the breathing rates of the subject change over time during the training session, we computed the moving average of the signals with 2 different window sizes (1 s and 20 s, respectively). Here, we denoted them as MA_1_ and MA_2_. During the slow breathing training, MA_1_ was used as the threshold to detect the peak for the first 20 s. After that, we set the threshold as Max (MA_1_, MA_2_). Once the points of maximum inhalation are identified, the breathing rate can then be calculated.

#### 3.3.2. Visualization

**Figure 5:**
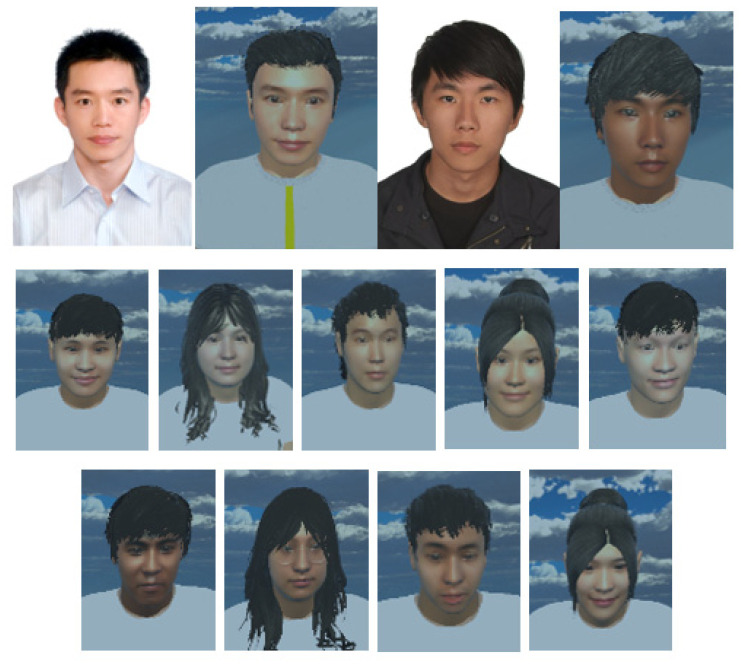
3D facial models based on photos of the users.

**Figure 6:**
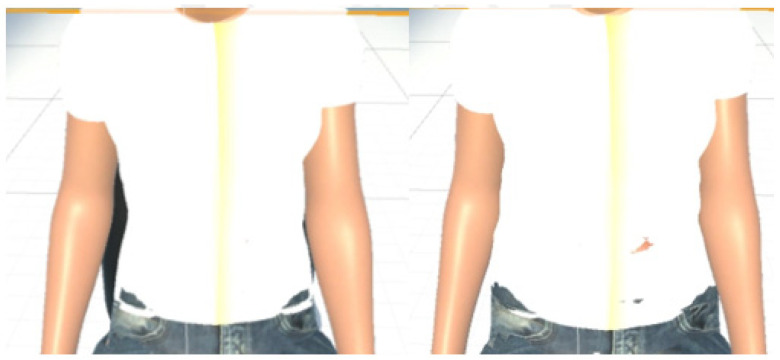
(**Left**) visualization of the exhalation (**Right**) visualization of the inhalation.

**Figure 7:**
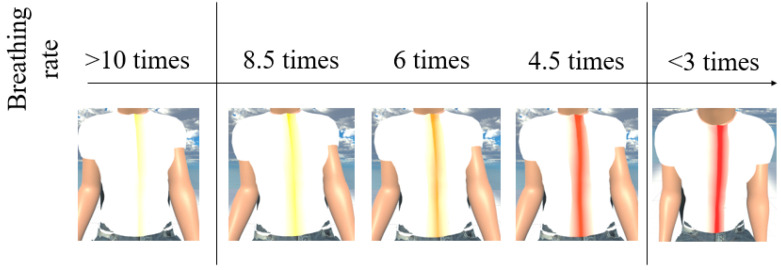
The visualization of changes of body temperature.

MCS (Morph Character System) (Daz 3D, Salt Lake City, UT, USA) [47] was used to generate the 3D human body model [47] in this work. In addition, to provide a more immersive experience to the user, we used FaceGen (Singular Inversions, Toronto, ON, Canada) [40] to create a realistic facial model in Unity3D based on a fontal face photo supplied by the user, as shown in Figure 5. The movements of the abdomen can be visualized according to the changes of pressure sensor data. In other words, the local maxima in the data correspond to the point of maximum inhalation while the minima in data indicate the point of maximum exhalation, as shown in Figure 6. Finally, in advanced meditation practices, such as g-tummo [45], an experienced meditator usually feels a rising body temperature along the central axis of their body. To simulate this changing of body temperature, we created a virtual line in the center of the body. The thickness and colors of this line change according to the breathing rates. Specifically, when the user breathes slower, this central line becomes thicker and its color changes from yellow to red. Figure 7 show the corresponding colors for different breathing rates.

### 3.4. User Preparation

**Figure 8:**
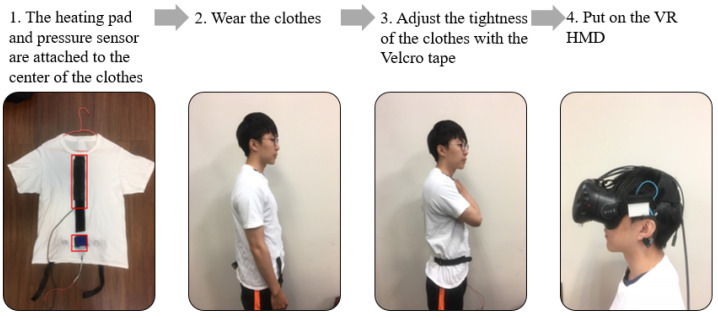
The user procedure of our study.

**Figure 9:**
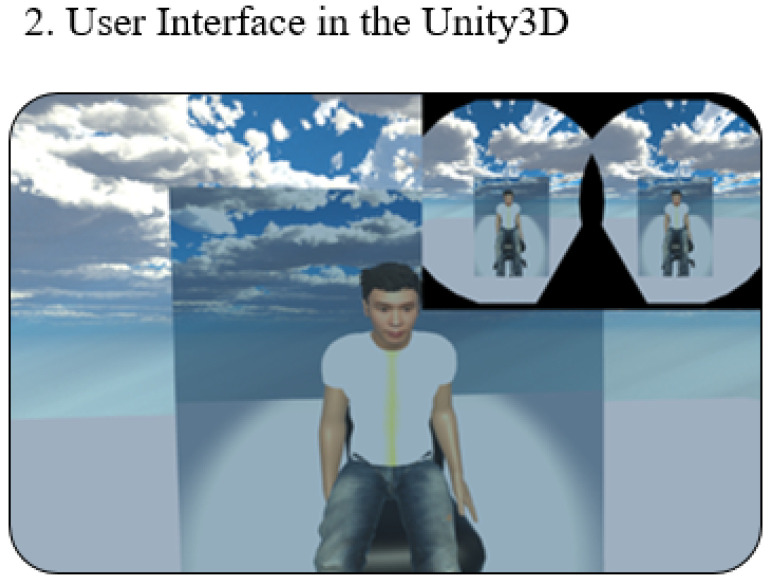
The VR interfaces.

To measure the breathing rate and provide tactile feedback, we designed a T-shirt with a pressure sensor and heating pad. Two pieces of Velcro tape were sewn onto the inner layers of the T-shirt and attached to heating pads and the pressure sensor (which was placed around the navel position). The user can adjust the Velcro tape to ensure the pressure sensor is tightly attached to the body, as shown in Figure 8. Once the user puts on the VR headset, he can see a 3D model that looks similar to himself sitting at the center of the screen, as shown in Figure 9. Typically, the preparation takes less than one minute.

## 4. Experiment and Result

While many studies have shown that slow breathing practice during meditation has many health benefits, many people find it difficult to maintain a regular slow breathing practice. To understand the usefulness of the proposed system, we recruited 20 university students and performed a set of experiments as detailed below. The subjects were divided into two groups (i.e., with and without feedback) which are referred to as ‘feedback’ and ‘control’ groups herein. As a feasibility study, the objective of these experiments is to answer the following questions:

1. Can the proposed system improve the breathing control of the user?
2. Does the proposed system have any effect on the meditation, in terms of the changes of EEG alpha and theta bands [28]?

In this study, we used ANOVA to measure the statistical difference between the control group and the feedback group. The significance threshold was set at 0.05. The details of our experiment setup are shown in Table 2.

### 4.1. Experiment Procedure

**Figure 10:**
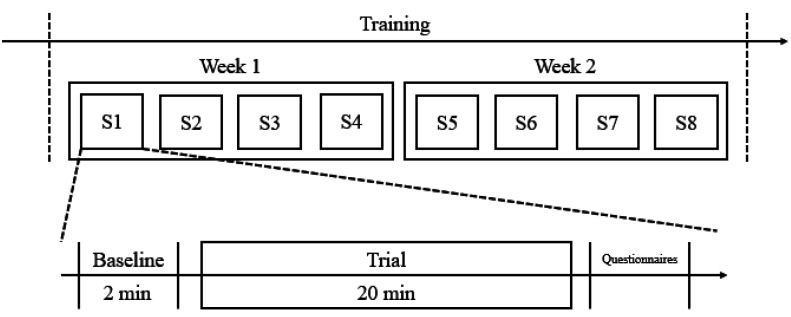
The experiment procedure of our study.

For each experiment, the subjects were first asked to take a 5 min rest and then prepare themselves as described in Section 3.4. A 2-minute baseline trial was conducted before a 20-minute session of slow breathing training. During the baseline trials, the subjects were advised to sit back and relax and breathe normally, while in each training session, the subjects were encouraged to slow down their breathing as much as possible. Once the training session is ended, the subjects were requested to fill in a questionnaire, as shown in Table 3. Eight training sessions were completed in two weeks. Specifically, four training sessions were executed in each week, as shown in Figure 10 (here, ‘S1′ refers to “session 1”; ‘S2′ refers to “session 2”, etc.).

### 4.2. Results

**Figure 11:**
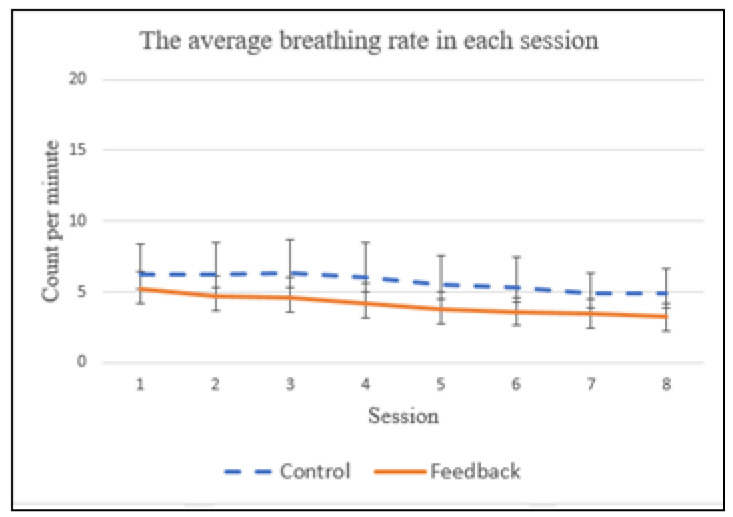
The average breathing rate in each session.

For the baseline trials, no statistical difference in the average breathing rates were observed between the control and feedback groups (*p* = 0.999). We found that the subjects improved their breathing rate over a two-week training period, for both control and feedback groups, as shown in Figure 11, which evidently shows that more practice sessions do improve breathing control skill. The average breathing rates for the control group were 6.24, 6.28, 6.32, 6.05, 5.48, 5.27, 4.93 and 4.93, from the 1st to 8th sessions, respectively. For the feedback group, they were 5.21, 4.73, 4.57, 4.15, 3.74, 3.65, 3.53 and 3.35, respectively. A significant statistical difference in the average breathing rate was found between the control group and feedback group (*p* = 0.0049) during the 2-week training period. We also conducted repeated statistical tests to examine if there was any statistical difference in breathing rates between the control and feedback groups for the same session (i.e., session one of the control group vs. session one of the feedback group; session two of the control group vs. session two of the feedback group, etc.). A statistical difference in the breathing rate has been observed between the control and feedback groups for the same session, except for the first session (*p* = 0.065).

The typical respiratory rate in humans is within the range of 10 to 20 breaths per minute. Many prior studies [3] have shown that a breathing rate lower than 4 CPM (counts per minute) [3] could alleviate stress, anxiety and depression. As shown in Figure 11, the subjects in the feedback group were able to already achieve an average breathing rate of 3.74 CPM at the 5th session, while the average breathing rate of the control group is still higher than 4 CPM (i.e., 4.93) at the end of the two-week training period. This suggests that the proposed multimodal biofeedback system is useful for improving breath control.

Given that the subjects might have different abilities in controlling their breathing rates, we were interested to explore whether the breathing rate of the subject changes significantly over the 20 min training period. To answer this question, we computed the standard deviation of breathing rates of each subject during the 20 min training period. Specifically, the average standard deviations in the control group were 1.0, 1.2, 0.98, 0.97, 0.99, 0.84, 0.82 and 0.78, from the 1st to the 8th session, respectively. For the feedback group, they were 0.68, 0.57, 0.58, 0.46, 0.46, 0.42, 0.47 and 0.44, respectively. A significant statistical difference in the average standard deviation of breathing rate has been found between the control group and feedback group (*p* = 0.001) during the two-week training. In addition, a statistical difference in the standard deviation of the breathing rate has been observed between the control group and feedback group for all eight sessions ((i.e., session one of the control group vs. session one of the feedback group; session two of the control group vs. session two of the feedback group, etc.). These results show that, guided by the multimodal feedback system, the subjects in the feedback group were able to control their breathing in a more uniform way during the training. In addition, we found that the average standard deviations gradually reduced over the two-week training period for both control and feedback groups. This once again proves the old saying “Practice makes perfect!”

**Figure 12:**
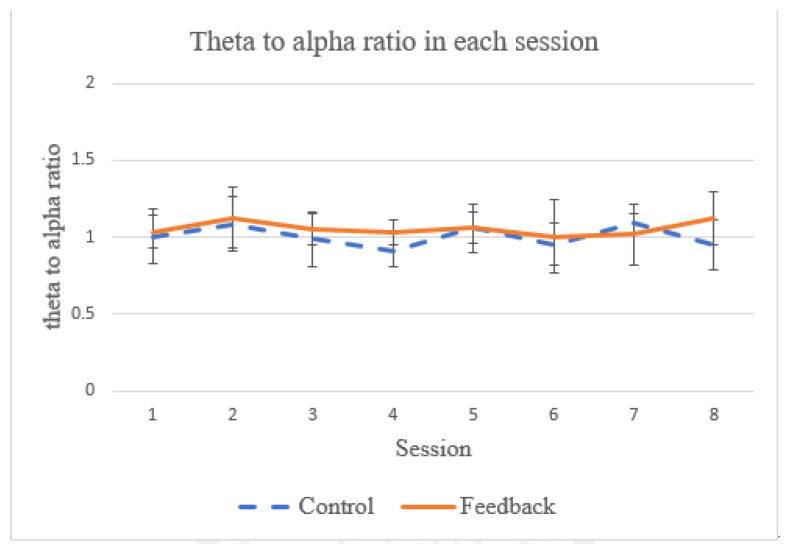
The average theta-to-alpha ratio in each session.

Theta and alpha power measurements from EEG data are often used when it comes to evaluating the success of meditation [48]. The ratio between the two power bands has been used as a reference to quantify respiratory effect on the meditation [28]. In particular, it has been shown that the theta power decreases (related to the state of concentration [49]) and the alpha power increases (related to the state of relaxation [49]) during meditation when focusing on breathing [50]. In this work, we also examine this ratio of theta-to-alpha power to see if our proposed system has any effect on the meditation (as compared to breathing training with no feedback). As shown in Figure 12, during the two-week training period, no statistical difference in this ratio was observed between the control group and feedback groups (*p* = 1.001). In addition, no statistical difference in theta-to-alpha ratio has been observed between the control and feedback groups for each individual session. This observation is in line with the findings reported by [28]. One possible explanation is that, while the biofeedback provides guidance for the breathing control strategy of the subject, the degree of ease to relax cannot be improved because the subject needs to pay attention to perceiving the biofeedback signals and, hence, the arousal cannot be significantly reduced.

**Figure 13:**
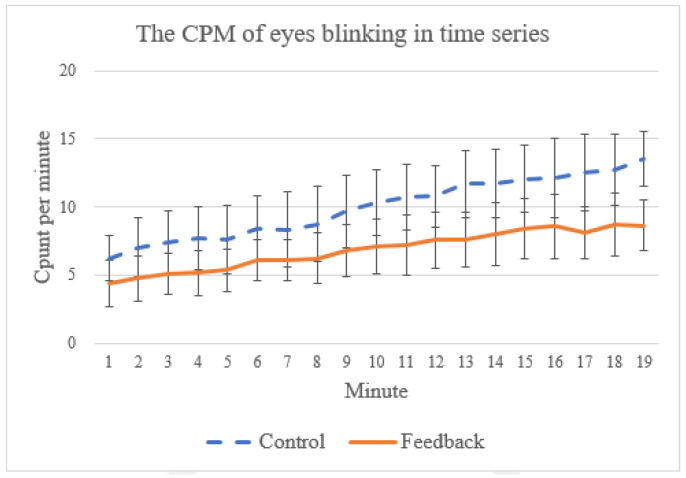
The average count per minute of eye blinking events from first to the 19th minute.

Finally, in practice, many people find it difficult practice meditation for a long time because they tend to fall asleep during meditation [50]. As shown in some prior work [51,52], the frequency of spontaneous eye blinking can be used as an indicator for the level of sleepiness. In EEG signals, eye blinks are typically characterized by peaks with relatively strong voltages [53,54,55]. In this work, we adopt a threshold-based method [53,54,55] which is a common way to detect eye blinks by classifying all events exceeding the threshold value (which, in this study, is based on a mean threshold algorithm from [55]) as eye blinks. To understand if our proposed system can help the subject stay awake, we count the number of eye blinking events during the training session, as the example (taken from one of the training sessions) in Figure 13 shows. In general, the subjects tend to become increasingly sleepy toward the end of the training (indicated by the increasing eye-blinking events) for both control and feedback groups. Nevertheless, we found a significant statistical difference in eye-blinking frequency between these two groups (*p* = 0.0009), which suggests that the proposed system can help the subjects reduce sleepiness during the training.

## 5. Discussion

In this section, we present a subjective assessment of the satisfaction of the users to the proposed system. In addition, we discuss the possible applications of the proposed multimodal VR system and some observations from our experiments.

**Figure 14:**
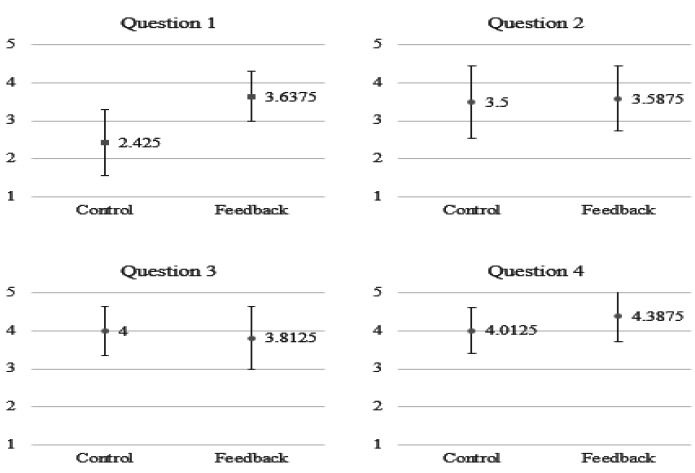

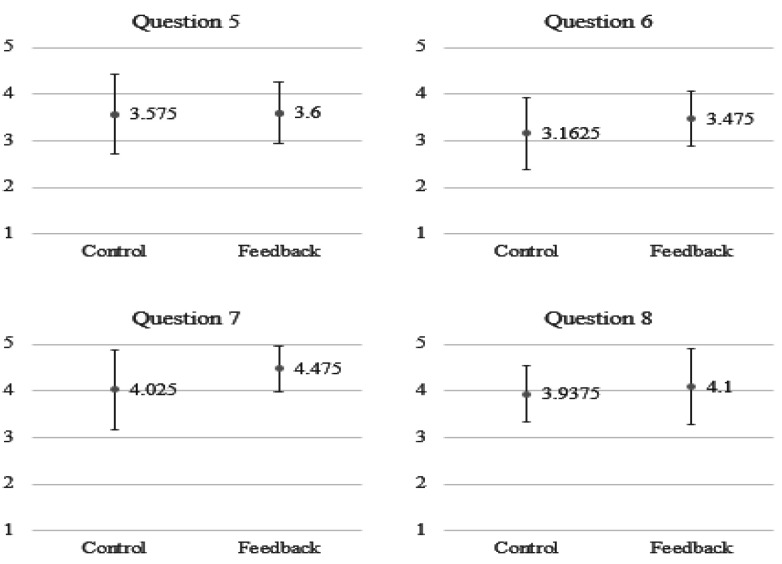
The average point for each item in the questionnaire.

To understand the satisfaction of the subjects when using the proposed system for breathing training, we asked the subjects to fill in a questionnaire, as shown in Table 3. We employed a 5-point Likert scale in which responders specified their level of agreement to a statement in five points: (1) Strongly disagree; (2) Disagree; (3) Neither agree nor disagree; (4) Agree; (5) Strongly agree. As shown in Figure 14, generally, the subjects in the feedback group have a stronger agreement for most of the questions. In particular, as indicated by their answers to the first question, they find it enjoyable to use such a multimodal feedback system for meditation training.

In this work, we propose a VR system with multimodal feedback for slow breathing training. The strength of multimodal interfaces lies in creating a stronger sense of presence by better mimicking reality [56]. The sensorial richness of multimodal environments translates into a more coherent experience of the virtual world and, therefore, the sense of being present in the virtual realm is stronger. The proposed VR-based multimodal biofeedback system could possibly be applied to other applications, such as sports [57] and healthcare [58]. For example, many prior studies have shown that providing visual feedback in VR is useful for improving gait for patients with Parkinson’s Disease (PD) [59,60]. Recently, some studies have suggested that tactile feedback can also alleviate Freezing of Gait (FOG) in PD [61]. One could possibly combine these two different types of feedback into the same VR environment, in the same way as our proposed system, for gait training PD patients.

In addition, while it might sound obvious, given that the users adjust their breathing control strategies based on feedback (including the changes of visualization and the temperature of heating pad), we find that it is important to ensure the feedback (according to the breathing rates) is clearly noticeable to the users. These are related to the design of GUI and the selection of actuators for such a feedback system. During our experiments (not detailed in this paper), we find that unclear feedback (e.g., indistinguishable color changes corresponding to the breathing rate) could significantly degrade the performance of such a feedback system.

## 6. Conclusions and Future Work

Numerous studies have reported the benefit elicited by meditation. It is commonly believed that breathing techniques are deeply intermingled with meditation. Regular practices of breathing control have been proven to have beneficial effects on health status, such as wellness, relaxation and stress reduction. However, many people find it difficult to maintain a regular practice of slow breathing with meditation due to various reasons, including lack of time, lack of progress feedback, and boringness. In addition, a long training period is usually required for benefit realization. To raise the user’s interest for a regular practice of slow breathing training, a virtual reality system with multimodal biofeedback is proposed in this paper. In our system, a pressure sensor is attached to the abdomen of the user to detect the breathing rate, which can then be visualized through VR as well as control the tactile feedback by changing the temperature of the user’s clothes. Through the two-week-long experiments, we found that the proposed multimodal feedback system is effective in improving slow breathing training in terms of achieving a slower breathing rate and smaller variations during the training process. However, we did not observe that such a respiratory feedback can facilitate lowering arousal in meditation. Finally, the users’ satisfaction with the proposed system was evaluated through questionnaires. Most of the users found it enjoyable to use the system for mediation training.

The current selected subject population in this work is limited. We plan to recruit more people from a wider population range to validate the suitability of our proposed system for people with various ages and health conditions (for example, some studies [62] showed that effects of biofeedback in children might be different from the effects of biofeedback in adults). In addition, in future experiments it will be useful to perform a questionnaire about the emotion/mood of the subjects, such as the Self-Assessment Manikin or Pick-A-Mood questionnaires, to understand the participant’s arousal. Moreover, in our future experiments, to further examine the effectiveness of respiratory biofeedback, we plan to include an additional control feedback placebo group, in which visual/tactile feedback is not paired to breathing (e.g., no visualization of abdomen movements due to the breathing). More advanced UI techniques will be also considered in the future to make the feedback more perceivable by the users.

## Figures and Tables

**Table 1:** Prior studies using respiratory feedback.

Study	Application
[24]	Effects of abdominal and thoracic breathing on breathing pattern components
[25]	Smartphone-based respiration training
[26]	The relationship between respiratory patterns and SCL (Skin conductance level)
[27]	Effect of respiratory feedback for patients with refractory epilepsy
[28]	Respiratory feedback through virtual reality

**Table 2:** Details of experiment setups.

Number of subjects	20 (10 for control group and 10 for feedback group)
Environment condition	Quiet room Room temperature: 23–26 °C
Inclusion criteria	No previous meditation experience Free of cardiac, pulmonary, metabolic and any other diseases that might cause autonomic nervous system dysfunction
Duration of experiments	Eight 20 min training sessions were carried out within 2 weeks (four sessions per week).
Time	Between 2 pm and 5 pm
Notes to the subject before the experiment begins	Do not eat one hour prior to the training Must have emptied one’s bowels or bladder before the experiment Five minutes of rest to be taken before the experiment starts The subject was informed about the average breathing rate in the previous training session and was requested to try improving it further.
Baseline trial	A two-minute baseline trial was conducted before each training session begins. The subjects was asked to be relaxed and sit with their back straight and arms hanging on both sides
Training session	Abdominal breathing is employed Subjects were asked to focus on slowing down their respiration rates but not holding their breath Subjects were asked to avoid excessive eye blinking during the training session

The average age of subjects is 20.6 years (SD = 2.3) for the control group (5 males and 5 females) and 22.0 (SD = 1.2) for the feedback group (5 males and 5 females). No significant statistical difference in gender and age between these two groups (*p* > 0.05).

**Table 3:** The questionnaires.

1. I find this breathing control experiment enjoyable
2. I was able to focus on my breathing without any distraction
3. During the experiment, I felt that my breathing rate was reduced
4. I think it is easy to use this system (clothes, sensors, etc.)
5. I do not think I need technical support to use this system
6. I will continue doing meditation training in the future
7. This system is suitable for meditation-related applications
8. I do not need to learn anything before I can use this system

## Data Availability

The original data used to support the findings of this study are available from the first author upon request.
